# 3D visualization of the lumbar facet joint after degeneration using propagation phase contrast micro-tomography

**DOI:** 10.1038/srep21838

**Published:** 2016-02-24

**Authors:** Yong Cao, Yi Zhang, Xianzheng Yin, Hongbin Lu, Jianzhong Hu, Chunyue Duan

**Affiliations:** 1 Department of Spine Surgery, Xiangya Hospital, Central South University, Changsha, 410008, China; 2 Center for Drug Delivery System, Shanghai Institute of Materia Medica, Chinese Academy of Sciences, Shanghai 201203, China; 3 Department of Sports Medicine, Research Centre of Sports Medicine, Xiangya Hospital, Central South University, Changsha, 410008, China

## Abstract

Lumbar facet joint (LFJ) degeneration is believed to be an important cause of low back pain (LBP). Identifying the morphological changes of the LFJ in the degeneration process at a high-resolution level could be meaningful for our better understanding of the possible mechanisms underlying this process. In the present study, we determined the 3D morphology of the LFJ using propagation phase contrast micro-tomography (PPCT) in rats to assess the subtle changes that occur during the degeneration process. PPCT provides vivid 3D images of micromorphological changes in the LFJ during its degeneration process, and the changes in the subchondral bone occurred earlier than in the cartilage during the early stage of degeneration of the LFJ. The delineation of this alteration was similar to that with the histological method. Our findings demonstrated that PPCT could serve as a valuable tool for 3D visualization of the morphology of the LFJ by providing comprehensive information about the cartilage and the underlying subchondral bone and their changes during degeneration processes. It might also have great potential for providing effective diagnostic tools to track changes in the cartilage and to evaluate the effects of therapeutic interventions for LFJ degeneration in preclinical studies.

Low back pain (LBP) is extremely common and is one of the most frequent symptom-related reasons for patients visiting physicians. More than 80% of the population will suffer from LBP at some point during their lives. LBP is also the leading cause of work-related disability, and causes a serious effect on people’s health worldwide^1^,^2^.

The lumbar facet joints (LFJ), located in the posterior aspect of the vertebral column, are the only true synovial joints between adjacent spinal levels, and have a highly specialized and complicated anatomical 3D structure^3^. The LFJ are in almost constant motion with the spine under certain stress, and simply wear out or become degenerated, leading to abnormalities in LFJ function^4^,^5^. In 1911, Goldthwait suggested that the LFJ were one source of LBP^6^. Ghormley also reported on “facet joint syndrome” (FJS) caused by LFJ degeneration in 1933, indicating that LFJ degeneration plays an important role in the development of LBP^7^,^8^. However, the detailed mechanisms underlying this disease have not been fully illuminated.

Describing the LFJ microstructure with intact organs and evaluating the micromorphological changes in the LFJ during degeneration would help us to gain further insight into this pathological process. Furthermore, acquiring such information requires a powerful imaging method for obtaining the detailed data on microstructural changes. However, previous studies relying on histological sectioning could not entirely yield the 3D morphology of the LFJ^9^,^10^. Clinically, CT could obtain more information about the LFJ structure, and its morphological changes during degeneration could also be detected. However, this method has poor contrast resolution of soft tissue, so the cartilage of the LFJ could hardly be detected^11^,^12^. While MRI can enable fine observation of the soft tissue structures, *in vivo* MRI measurement of the morphological changes of the LFJ has already been described^13^. However, the imaging resolution of MRI remains at the millimeter scale, without any contrast agent used^11^,^12^,^13^. Identification of the subtle changes in small LFJ and rendering of the 3D morphology of the thin layer cartilage on the surface of LFJ using MRI can sometimes be limited.

Phase contrast imaging (PCI), based on synchrotron radiation, is an innovative method. In addition to producing projection images, PCI can be combined with tomographic (CT) to form phase contrast tomography (PCCT), which can determine the 3D morphology of the inner structures of bio-samples^14^. PCI imaging of objects depends on the refraction of the X-rays in matter, and it is quite different from conventional X-ray imaging, which is based on the differential X-ray attenuation by the constituents of objects. The phase shift is also 1000 times greater than the absorption of light elements in hard X-ray regions, indicating that PCI is more sensitive to density variation in samples, and it provides significant phase contrast information about weak absorption objects, such as soft tissue, or objects with low atomic number (Z) materials^15^,^16^,^17^.

Previous studies have mentioned that a variety of PCI techniques have been developed, including interferometer imaging (II), diffraction-enhanced imaging (DEI), grating-based imaging (GI) and propagation phase contrast imaging (PPCI)^18^,^19^,^20^. Among all these imaging methods, PPCI is the simplest to implement, requiring only a double-crystal monochromator without the need for additional complex optical components^21^,^22^. PPCI can provide excellent phase contrast information about soft tissue, such as the liver, brain and spinal cord vessels, and cartilage, especially in the human knee joints^23^,^24^,^25^,^26^,^27^,^28^,^29^. The 3D morphology of small objects, even individual cells, can be obtained with high spatial and contrast resolution using this method^30^. However, the application of PPCI combined with CT for 3D imaging of the LFJ has not been conducted. In addition, the morphological changes of the LFJ during the degeneration process have also not been investigated using this imaging technique.

In the present study, propagation phase contrast micro-tomography (PPCT), using synchrotron radiation, was applied for detection of the 3D morphology of the LFJ, emphasizing joint cartilage investigation *in vitro*. After an LFJ degeneration model was generated in rats, this method was used to obtain micro-tomography of the subtle structure changes in the LFJ during the degeneration process, which aided in exploring the mechanisms underlying this disease.

## Results

### LFJ Cartilage

**Figure 1:**
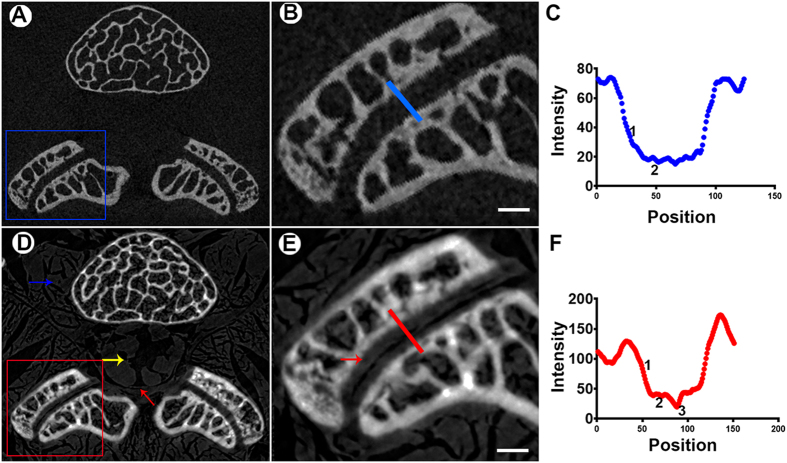
Comparison of PPCT with μCT in cartilage visualization. (**A**,**D**) Transverse sections acquired using PPCT and μCT. The PPCT images show markedly superior soft tissue contrast and delineation as well as cartilage depiction, compared with μCT. Blue arrows indicate the paravertebral muscles. The yellow arrow indicates the nerve tissue. (**B**,**E**) Enlarged images of the LFJ from A and D clearly demonstrate the structure of the LFJ, particularly the cartilage (red arrows). (**C**,**F**) The lines across the LFJ from B and E. Detailed information about the structure of the cartilage and the space were detected by PPCT, compared with μCT. Bar = 500 μm.

**Figure 2:**
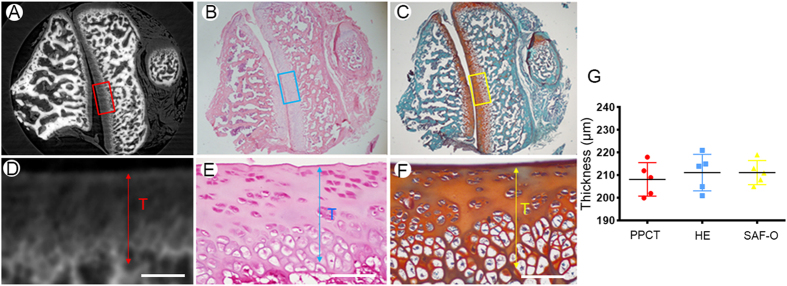
Comparison of PPCT with histological methods for cartilage visualization. (**A**) Transverse sections detected using PPCT, (**B**) HE, and (**C**) safranin-O staining. (**D**–**F**) Higher magnification of the area marked on the corresponding images with rectangular boxes. (**G**) Thickness measurements using these three tools. Bar = 100 μm.

**Figure 3:**
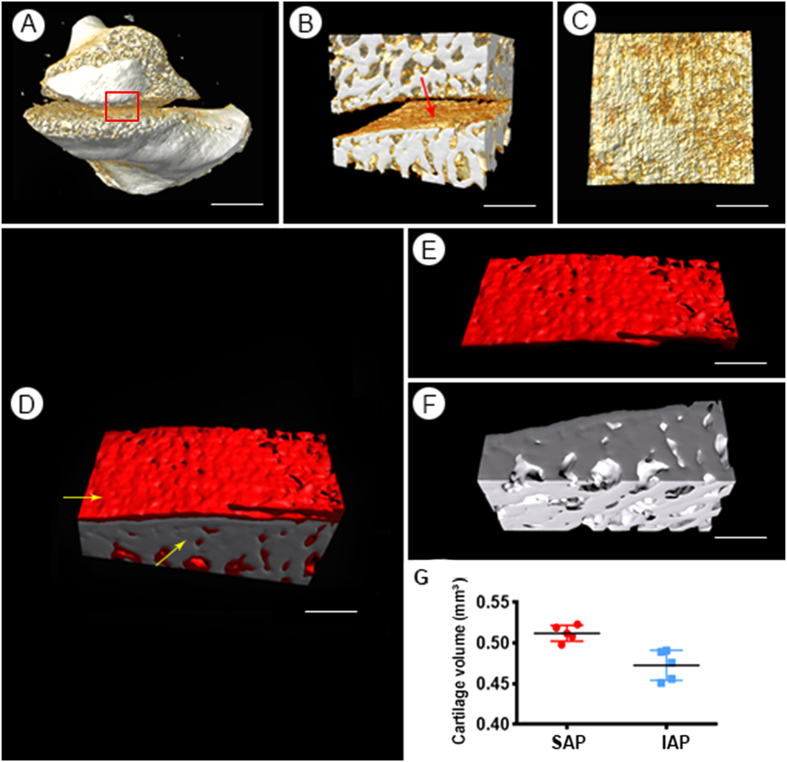
3D visualization the LFJ. (**A**) The 3D morphology of the LFJ detected by PPCT; (**B**) ROI of the LFJ. (**C**) The facet surface of the SAP. (**D**) 3D pseudo-color map of the SAP; (**E**,**F**) the morphology of the cartilage and subchondral bone were separated and could be vividly visualized. (**G**) The cartilage volume of the SAP and IAP. (**A**) Bar = 2.5 mm. (**B**,**C**) Bar = 1 mm. (**D–F**) Bar = 500 μm. ROI: region of interest; SAP: superior articular process; IAP = inferior articular process.

The reconstructed sections extracted from micro-tomography (μCT) and PPCT are shown in Fig. 1. The border and structure of the muscle tissue located at the lumbar vertebra with high contrast are clearly depicted. Additionally, the demarcation of nerve tissue, as well as the layer of endorachis in the vertebral canal, was also readily observed (Fig. 1D), which are invisible on μCT (Fig. 1A). As an exclusive finding, PPCT vividly revealed the cartilage structure and delineation of its surface (Fig. 1E), which could not be distinguished on μCT transverse sections. The line across the LFJ marked in Fig. 1B,E depicts the local details of the LFJ detected by PPCT and μCT (Fig. 1C,F). The structure of the underlying subchondral bone (Fig. 1F-1) and cartilage (Fig. 1F-2) could be distinguished from the joint gap (Fig. 1F-3) on the PPCT transverse section, while these structures were difficult to observe on the μCT images (Fig. 1C). To confirm the structure of the thin layer of the cartilage, we performed a comparative study between the PPCT slice and histological staining of the same specimens. Both of these tools provided excellent depiction of the LFJ structure, particularly the cartilage. The visible cartilage showed sharp surface demarcation on PPCT, as well as with HE and safranin O–Fast green staining (Fig. 2A–C), which allowed for thickness measurements. No significance differences in cartilage thickness were detected among these methods (Fig. 2D–G).

### 3D visualization of cartilage in un-sectioned LFJ tissue

To visualize the cartilage in a volumetric manner, the 3D reconstruction of the transverse slice images was performed. The 3D microstructure of the LFJ without tissue sections was first rendered using PPCT and is shown in Fig. 3A. Based on the images obtained, the LFJ exhibited distinct features, the articular cartilage covered the opposing surfaces of the facets, and a layer of subchondral bone could be readily observed with high resolution. To trace the morphology of the cartilage covering the superior articular process (SAP), the region of interest (ROI), marked with square features in Fig. 3A, was selected and showed in Fig. 3B. The cartilage of the LFJ could be vividly visualized and demonstrated in multiple perspectives (Fig. 3C). Due to the difference in the density of the cartilage and the surrounding background tissue, the cartilage and subchondral bone structure could be segmented and defined manually, and each was assigned a different color coding to enhance the observation, effectively resulting in detailed detection of its microstructure on various views (Fig. 3D–F). The cartilage volume of the SAP was 0.512 ± 0.0035 mm^3^, while that of the inferior articular process (IAP) was 0.4726 ± 0.0175 mm^3^. The cartilage volume of the SAP was slightly larger than that of the IAP (Fig. 3G).

### 3D visualization of the LFJ after degeneration

**Figure 4:**
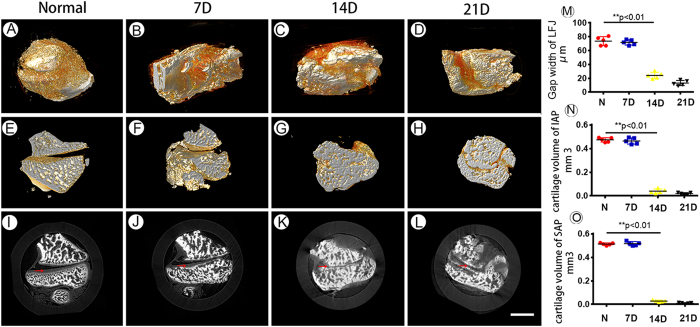
The 3D morphological changes of the LFJ during the degeneration process. (**A–D**) 3D images of the normal LFJ and degenerated LFJ at different stages, 7, 14 and 21 days after injection with MIA. (**E–H**) Sagittal views of the LFJ at different stages after injection with MIA. (**I–L**) The corresponding transverse slice images of the LFJ. (**M–O**) The joint gap width and cartilage volume of the IAP and SAP. Bar = 2 mm.

The 3D morphological changes in the LFJ during the degeneration process induced by monosodium iodoacetate (MIA) detected by PPCT and are displayed in Fig. 4A–D. To assess objectively and quantitatively the structural modifications of the LFJ, the gap width of the LFJ was calculated (Fig. 4E–H). Significant decreases in the gap width were detected 14 days after injection with MIA. The LFJ became fused, the gap width narrowed, and osteophytes were observe at the margin of the LFJ at 21 days (Fig. 4H). However, the gap width showed no significant decrease in the early stage, at 7 days after injection with MIA (Fig. 4M). To quantify further the progression of the LFJ degeneration process, the volume of the cartilage that covers the LFJ was calculated. Loss of the cartilage covering the facet joint could be vividly observed from the corresponding slice images, showing progression of the degeneration process (Fig. 4I–L). At 14 days, the cartilage volume of both the SAP and IAP was significantly decreased, suggesting that loss of cartilage was the prominent feature of LFJ degeneration. However, in the early stage during the first weeks after injection with MIA, the morphological changes in the cartilage volume were not obvious on the PPCT images (Fig 4N,O).

### Cartilage and subchondral bone changes in the early stage detected by PPCT

**Figure 5:**
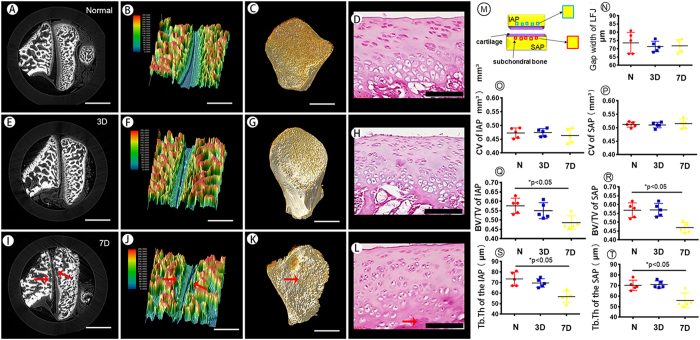
The structural changes in the cartilage and subchondral bone in the early stage during LFJ degeneration. (**A**,**E**,**I**) Transverse slice images of the LFJ both normal and at 3 and 7 days after injection with MIA. (**B**,**F**,**J**) 3D color-coding maps of the LFJ during the corresponding stages. (**C**,**G**,**K**) The 3D morphology of the subchondral bone in the SAP during the corresponding stages after injection with MIA. (**D**,**H**,**L**) The histological and morphological changes in the LFJ during the degeneration process in the early stage. (**M**) The scheme depicting the image analysis process for the subchondral bone. (**N**) The morphological change in the joint gap width of the LFJ during different stages. (**O–T**) The cartilage volume (CV), BV/TV and Tb.Th of the SAP and IAP at different stages after injection with MIA. (**A**,**C**,**E**,**G**,**I**,**K**) Bar = 1 mm. (**B**,**F**,**J**) Bar = 500 μm. (**D**,**H**,**L**) Bar = 100 μm.

During the early stage of the degeneration process, the subtle structural modifications in the cartilage and subchondral bone were detected by PPCT. The morphological alterations of the cartilage and the subchondral bone are vividly delineated on the PPCT (Fig. 5A,E,I). To optimize the depiction of the cartilage and subchondral bone, 3D color-coding maps were applied (Fig. 5B,F,J). There were no significant differences detected in the joint gap width or cartilage volume (CV) of the inferior and superior facets on the 3rd day of degeneration (Fig. 5N–P). The measurement of the BV/TV (trabecular bone volume per tissue volume), and the Tb.Th (trabecular thickness) of the subchondral bone of the LFJ showed no differences in either IAP or SAP at this stage, compared with the normal LFJ (Fig. 5Q,R). However, at 7 days, a lesion appeared in the subchondral bone area (red arrow) that could be vividly visualized, and it was more noticeable than the cartilage, compared with 3 day group and the normal LFJ. Additionally, the 3D surface of the subchondral bone could be rendered, and the microstructure of the subchondral bone was altered during the early stage after MIA injection and was characterized by severely elevated and depressed features (Fig. 5C,G,K). Furthermore, the measurements of the BV/TV and Tr.Th of the subchondral bone in the IAP and SAP both demonstrated significant decreases at 7 days. However, the joint gap width and the volume of the cartilage covering the apposed surfaces of the facets showed no significant changes at this stage. The pathological changes on PPCT images were confirmed by histological staining of the cartilage and the subchondral bone area during this process (Fig. 5D,H,L).

## Discussion

LFJ degeneration has been considered to be a main contributing factor to LBP. However, the detailed mechanism underlying this process has not been fully illuminated. Rodents have frequently been used for experimental investigation of the mechanism of LFJ degeneration, and various rat models of LFJ degeneration have also been established^10^. In this study, we successfully generated a LFJ degeneration model by injection of rats with MIA, which has already been described in previous studies^31^,^32^. We used PPCT, based on synchrotron radiation, to visualize the 3D morphology of the LFJ both at normal status and their 3D structural changes during the degeneration process. This preclinical study demonstrated that PPCT was capable of visualizing the LFJ in 3D. And it accurately detecting the signs of LFJ degeneration, with excellent representation of the cartilage and subchondral bone structure. The subtle morphological alterations in the rat LFJ observed by PPCT were also correlated well with those observed using histologic methods. Additionally, the changes in cartilage and subchondral bone could be assessed quantitatively using PPCT, which was impossible with histologic methods. To our knowledge, there have also been no previous studies of the application of PPCT for quantitative measurement of cartilage and subchondral bone of the LFJ during degeneration processes in rat models.

Detailed morphological changes in the LFJ in various diseases largely rely on histological studies, which are the gold standard for tissue characterization^9^,^10^. However, histology provides only partial information about the LFJ, the integrity of specimen has been destroyed. While the μCT can preserve the 3D nature of specimens, soft tissue, such as the cartilage structures, are not perceptible with it^11^,^12^. The accurate evaluation of LFJ morphology with this technique would be difficult due to its limitation.

PPCT presents high soft tissue contrast and provides more detail with clear distinction of the muscular, neural and cartilage structures and even of individual cells^21^,^29^,^30^. Additionally, we can obtain 3D quantitative measurements and analyze the morphological changes in the cartilage of small animals, which are highly remarkable with this method and suggest a new dimension for accurately studying morphological changes in LFJ structures during the degeneration process. Moreover, PPCT data are available from intact specimens with potential cellular resolution, allowing for virtual sectioning with multiple views. In our study, the transverse virtual sectioning of the LFJ matched well with histological data given the high resolution, and it proved to be a powerful tool to distinguish the subtle changes between healthy and degenerated specimens.

Furthermore, a 3D color-coded map on PPCT was used to evaluate the morphological changes in the cartilage and subchondral bone of the LFJ; interestingly, we found that the subtle changes in the subchondral bone occurred earlier than in the cartilage during degeneration of the LFJ in the early stage. This pathogenesis of the LFJ, as well as the structural modification during the degeneration process, was consistent with that observed in human osteoarthritis (OA) pathology^8^. The quantitative measurement of cartilage and subchondral bone on the PPCT images also showed good agreement with the morphological observations. As the literature has described, as a morphological and mechanical unit, the subchondral bone provides linkage of the hyaline cartilage and cancellous bone, and it plays an important role in attenuating the impact force on the facet joint^33^,^34^,^35^,^36^. If morphological alteration of the subchondral bone occurs, the loading on the LFJ and its function will be affected^37^,^38^. The observations of our current study suggested that the microstructural changes in the subchondral bone might play important roles in the development of LFJ degeneration, and further studies to illuminate its role will be needed.

A unique strength of the current study was the application of PPCT for 3D visualization of the LFJ with intact organs, which could delineate the changes during disease progression. It could be extremely interesting and important to conduct a treatment intervention using this animal model and to analyze its effects using PPCT. Despite the superior image quality of PPCT provided by the high monochromatic synchrotron radiation light source, it nevertheless posed some limitations. Because of the limited field of the current PPCT facility, it was impossible to perform *in vivo* visualization of the 3D morphology of the LFJ in rats. Therefore, the intact LFJ had to be harvested and cut into a proper size to fit the image files of the imaging facility. Our ongoing study aims to transform this *in vitro* study to *in vivo* testing using a small mouse model, and in this situation, each mouse could serve as its own control. Additionally, radiation exposure is a concern in any clinical examination. The radiation dose for this experiment should be evaluated according to the clinical criteria. The assessment of LFJ cartilage lesions by PPCT could be made more meaningful by comparison with clinical imaging modalities.

Our findings demonstrated that the LFJ degeneration model was successfully induced by MIA. Overall, this study developed and validated a novel and valuable tool that allows 3D visualization of the morphology of the LFJ by providing comprehensive information about the cartilage and the underlying subchondral bone on PPCT. Application of this technique has tremendous potential for the evaluation of morphological changes in the LFJ during the degeneration process. This approach might also have great potential for providing an effective diagnostic tool to track changes in the cartilage of the LFJ and to evaluate the effects of therapeutic interventions for LFJ degenerative disease in preclinical studies.

## Method and Materials

### Animals

All of the experimental protocols were approved by the Animal Ethics Committee of Central South University. All of the procedures were performed in accordance with the approved guidelines. Male Sprague-Dawley (SD) rats (weight 200–220 g), obtained from the Animal Center of Central South University, were used for this experiment. The animals were housed under standard laboratory conditions in a temperature-controlled room with a normal 12-h light/dark cycle and with free access to food and water.

### Induction of LFJ degeneration model

LFJ degeneration was induced experimentally, as described previously^31^. In brief, the rats were anesthetized with an intraperitoneal injection of 1% pentobarbital sodium at a dose of 40 mg/kg. Subsequently, the rats were placed in the prone position, and a 2-cm posterior midline skin incision was made. The left para-spinal muscles were retracted to expose the left L4/L5 facet joint. A 34 G needle attached to a 5-μL microsyringe (SGE-Germany GmbH, Weiterstadt, Germany) was inserted into the articular cavity.

The degeneration group (n = 20) was administered a single intraarticular injection of monosodium iodoacetate (MIA; Sigma, St. Louis, MO, USA) at a dose of 0.1 mg in 5 μl sterile saline. This dose of MIA was optimized and was the most appropriate to mimic the LFJ degeneration process. The control group (n = 5) was administered a single intraarticular injection of an equal volume of sterile saline. Then, the wound was sutured. Five rats were not specially treated and served as normal controls.

### μCT

The structure of the LFJ were evaluated by μCT scanning. The freshly dissected L4/L5 LFJ was immediately fixed in 10% formalin and was kept in a tube for μCT imaging analyses in the Central South University imaging core facility, using a μCT (Skyscan 1172). The scanner was set at a voltage of 55 kVP, a current of 145 μA and resolution of 3.4 μm. Image reconstruction software (NRecon, version 1.6) was used to generate 2D transverse slice imaging and 3D imaging.

### PPCT

**Figure 6:**
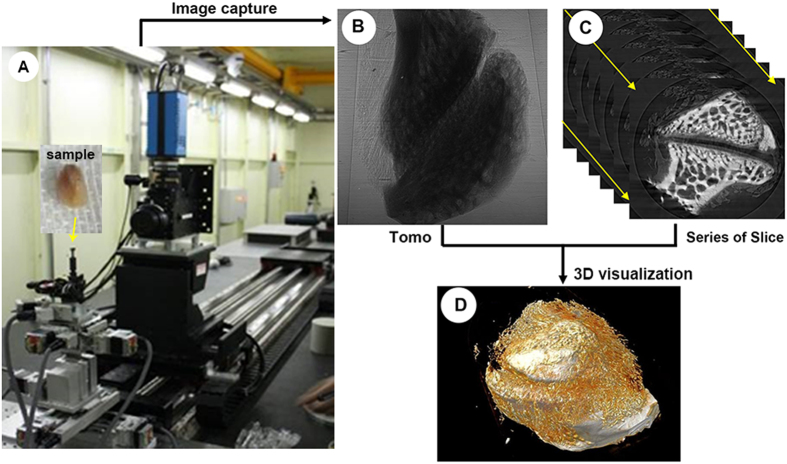
A picture of the imaging apparatus at BL13 W1 beamline of SSRF and a depiction of the scheme for the 3D imaging process using this technique. (**A**) The sample was mounted on the multidimensional sample stage, and a high-resolution CCD was used to capture the tomo-projection images of the LFJ. (**B**) Tomo images. (**C**) A series of 2D slice images. (**D**) 3D visualization of the LFJ.

PPCT scanning was performed to examine structural alterations in the LFJ cartilage and subchondral bone architecture region, using a BL13W1 beam line at the Shanghai synchrotron radiation facility (SSRF) in China (Fig. 6A). To obtain high phase contrast information, the scanned parameters were optimized and set to conditions with 15 keV of photon energy and voxel resolution of 7.4 μm, which is the same as that of μCT, and the sample-to-detector distance (SDD) was adjusted to 15 cm. The specimen was mounted at the sample stage and was rotated 180° around its central axis, resulting in 720 tomo-projections. The total projected images were transformed into transverse slice sections using the fast slice reconstruction software compiled by the BL13W1 experimental station of SSRF, based on the FBP algorithm. Subsequently, the series of 2D transverse slices were rendered for 3D visualization using VG Studio Max software (version 2.1, Volume Graphics GmbH, Germany) (Fig. 6B–D).

### Histological testing

After PPCT scanning, we fixed the LFJ segment in 10% buffered formalin for 48 hours, decalcified it in 10% ethylenediamine tetraacetic acid (pH 7.4) for 21 days and embedded it in paraffin. Four–μm–thick sagittal oriented sections of the LFJ segment were processed for hematoxylin and eosin (HE) and safranin (SAF) O–Fast green staining using the standard protocols^10^.

### Image analysis

The thickness of the cartilage and the gap width of the LFJ were measured using the Image-Pro Plus computer program (version 6.0, Media Cybernetics, Bethesda, MD). After PPCT scanning, the cartilage in the LFJ surface could be extracted using a gray value-based segmentation algorithm method commercially available in VG studio Max software. The 3D gray scale mapping of transverse slice imaging could also be displayed by this technique. In addition to qualitative analysis, we defined the region of interest (ROI) to cover the cartilage and the underlying subchondral bone compartment. The cartilage volume, the BV/TV (trabecular bone volume per tissue volume), and the Tb.Th (trabecular thickness) of the subchondral bone area per specimen in five different areas in each group were calculated.

### Statistical analysis

All of the data in the text and figures are presented as the mean ± standard error of the mean (SEM). All of the data analyses were performed using SPSS analysis software (SPSS, version 15.0, Inc.). The comparisons of cartilage volume and microarchitecture of the subchondral bone among different groups were performed using multifactorial ANOVA. When ANOVA indicated overall significance of the main effects without interaction between them, the differences between individual time points and sites were assessed by post hoc tests. A value of P < 0.05 was regarded as significant.

## Additional Information

**How to cite this article**: Cao, Y. *et al.* 3D visualization of the lumbar facet joint after degeneration using propagation phase contrast micro-tomography. *Sci. Rep.* **6**, 21838; doi: 10.1038/srep21838 (2016).
