# Corrigendum: 3D visualization of the lumbar facet joint after degeneration using propagation phase contrast micro-tomography

**DOI:** 10.1038/srep24511

**Published:** 2016-05-09

**Authors:** Yong Cao, Yi Zhang, Xianzhen Yin, Hongbin Lu, Jianzhong Hu, Chunyue Duan

Scientific Reports 6: Article number: 21838;10.1038/srep21838 Published online: 02242016; updated: 05092016

The original version of this Article contained a typographical error in the spelling of the author Xianzhen Yin, which was incorrectly given as Xianzheng Yin. This has now been corrected in the PDF and HTML versions of the Article.

